# On the nature and function of organizers

**DOI:** 10.1242/dev.159525

**Published:** 2018-03-01

**Authors:** Alfonso Martinez Arias, Ben Steventon

**Affiliations:** Department of Genetics, University of Cambridge, Cambridge CB2 3EH, UK

**Keywords:** Axial organization, Spemann, Body plan, Neural induction, Organizer, Vertebrate embryo

## Abstract

Organizers, which comprise groups of cells with the ability to instruct adjacent cells into specific states, represent a key principle in developmental biology. The concept was first introduced by Spemann and Mangold, who showed that there is a cellular population in the newt embryo that elicits the development of a secondary axis from adjacent cells. Similar experiments in chicken and rabbit embryos subsequently revealed groups of cells with similar instructive potential. In birds and mammals, organizer activity is often associated with a structure known as the node, which has thus been considered a functional homologue of Spemann's organizer. Here, we take an in-depth look at the structure and function of organizers across species and note that, whereas the amphibian organizer is a contingent collection of elements, each performing a specific function, the elements of organizers in other species are dispersed in time and space. This observation urges us to reconsider the universality and meaning of the organizer concept.

## Abstract (precis)

**Summary:** This Review re-evaluates the notion of Spemann's organizer as identified in amphibians, highlighting the spatiotemporal dispersion of equivalent elements in mouse and the key influence of responsiveness to organizer signals.

## Introduction

In the context of an embryo, an ‘organizer’ refers to a group of cells that harbour the ability to instruct fates and morphogenesis in surrounding cells, steering their development into specific organs and tissues (Anderson et al., 2016). As a result, organizers can position specific tissues and organs relative to each other. The term ‘organization centre’ was first introduced by Hans Spemann (Spemann and Mangold, 1924) in his interpretation of a classic experiment in which he and Hilde Mangold showed that the blastopore lip of the early gastrula of the newt *Triturus taeniatus* had the ability to cause the formation of a full axis when transplanted onto the opposite side of a similarly staged embryo of *Triturus cristatus*, a different unpigmented species. This experiment followed earlier observations by Spemann and others on the appearance of ectopic axes in transplantation experiments with amphibian embryos (for historical perspectives, see De Robertis, 2006; Gerhart, 2001). However, unlike the earlier studies, the 1924 report could discern between the host and the graft by pigment differences, which revealed the important point that the ectopic tissue developed from the host tissue.

Over the years, this observation – and the existence of the so-called Spemann organizer (see Glossary, Box 1) – has been confirmed by many different experiments and has become a pillar of developmental biology (De Robertis et al., 2000; Stern, 2001). Importantly, C. H. Waddington expanded the notion of the organizer to birds and mammals in experiments in which he transplanted a piece from the leading edge of the primitive streak of chicken, duck and rabbit embryos into early chicken embryos and observed a duplication of the anteroposterior axis of the host (Waddington, 1932, 1954). The tissue he was transplanting contained a structure known as the node (see Glossary, Box 1), and led to a conceptual association between the node and the organizer. An important landmark in all these experiments is the emergence of a secondary nervous system at the beginning of the inductive process, and for this reason the function of the organizer is often associated with a process called ‘neural induction’ (De Robertis and Kuroda, 2004; De Robertis et al., 2000; Stern, 2005).

Box 1. Glossary **Fate maps.** These can be defined as ‘projections of advanced developmental stages of an organism back to an earlier stage’ (Lane and Sheets, 2006) and, as such, are tightly associated with the notion of a body plan. Cell fates are mapped with reference to the three principal body axes. The anteroposterior (AP) axis defines the longitudinal organization of the body, with the head at one end and the tail at the other. The dorsoventral (DV) axis lies perpendicular to the AP and defines the arrangements of germ layer derivatives. The left-right (LR) axis distributes bilateral asymmetries.

**Gastrulation.** An organized sequence of cell movements and rearrangements that generate the three classical germ layers. Gastrulation results in a dramatic topological transformation out of which emerges a recognizable structure with axes and primordia for tissues and organs. In amniotes, gastrulation can be separated into primary and secondary gastrulation. Whereas a classical view suggests that gastrulation ends when the node appears, a more modern view deems the events associated with axial extension as a continuation of gastrulation. In both avian and mammalian embryos, axial extension occurs after the emergence of the node, which thus provides a landmark to separate two processes. Here, we suggest and shall use the term ‘primary gastrulation’ for the ingression/egression events that precede the node and ‘secondary gastrulation’ for their continuation during axial extension.

**Node.** A group of cells located at the leading edge of the primitive streak of amniote embryos that comprises mesodermal and endodermal derivatives, and also plays a specific and conserved function in the determination of bilateral asymmetries (Blum et al., 2007; Hirokawa et al., 2006; Lee and Anderson, 2008; Viebahn, 2001).

**Spemann organizer.** A multicellular structure situated above the developing blastopore lip in the gastrula stage amphibian embryo that when transplanted to a different but specific area of the embryo (the opposite pole) is able to: (1) induce an ectopic neural plate; (2) act as the source of cells for the prechordal plate and the notochord; and (3) promote convergence and extension movements in host cells (Gerhart, 2001; Harland and Gerhart, 1997). It was noted by C. Stern that Spemann had initially referred to ‘a piece of embryonic tissue that creates an “organization field” of a certain [axial] orientation and extent, in the indifferent material in which it is normally located or to which it is transplanted’, and that ‘this concept embodies both induction and patterning: the grafted cells change the fate of the responding tissue, and also generate a coherent (“organized”) set of structures’ (Stern, 2001).

A strict definition requires that for a group of cells to be an ‘organizer’, in Spemann's sense, it should be capable of inducing a neural plate and a complete body axis, as well as promoting movements of convergence and extension on adjacent groups of cells. Such an organizer can be identified by experiments in which a putative organizer is transplanted and grafted to a new position where its effects on host tissue are tested. However, it is important to consider three features associated with the interactions between graft and host: (1) a fate map (see Glossary, Box 1) of the host tissue at the time of the experiment, to understand the changes in fate that might result from the interaction and that are central to the function of the organizer; (2) knowledge of the degree of determination of the donor tissue to ensure that the transplanted cells retain their fate and organizing ability upon transplantation; and (3) knowledge of the competence state of the responding tissue, i.e. its ability to respond to the inducing tissue, so that we can assess the actual effect of the graft on the development of the host. In addition, understanding an organizer requires insights into the molecular nature of the events underlying the induction process. A molecular analysis of Spemann's organizer in *Xenopus*, for example, showed that it acts as a source of signalling molecules and that its action simply reflects the activity of these molecules. For the most part, these molecules are inhibitors of Wnt, Nodal and BMP signalling which account for all the effects of the dorsal lip (reviewed by De Robertis et al., 2001; Harland and Gerhart, 1997).

The organizer concept is a recurrent theme in the description of many developmental events (Anderson et al., 2016). Even in *Drosophila*, the dorsoventral and anteroposterior compartment boundaries of the wing disc are often referred to as ‘organizers’ (Diaz-Benjumea and Cohen, 1995; Neumann and Cohen, 1997; Struhl and Basler, 1993; Zecca et al., 1995). However, for a concept to be useful, it needs to have a robust definition that is applied with consistency and logic. This is particularly important when comparing development across species, as the timing of events and the molecular nature of the processes underlying them vary from organism to organism.

Here, we take an in-depth look at the notion of ‘organizer’, in the sense of Spemann's original definition in the amphibian experiments, and find that when translated to other experimental systems inconsistencies emerge that need to be confronted. For example, we find that there is little evidence for functional or structural homology between the node and the Spemann organizer. We also discuss how the specificity of the inductive event relies on the state of the host as well as on the activity of the organizer itself. Furthermore, we highlight how differences in the timing of gastrulation (see Glossary, Box 1) and in the growth rates of progenitor populations in different organisms make a universal functional relationship untenable. We conclude that the Spemann organizer is a specific collection of cellular elements, each endowed with defined functionalities. In amphibians, which undergo a relatively rapid rate of early embryogenesis, these functionalities are located within a single ensemble that when transplanted can elicit all the characteristics associated with an organizer. However, in other species, for example the mouse, equivalent elements are spatially and temporally dispersed such that no single tissue can be described as being homologous to the amphibian organizer. Our considerations highlight the importance of the competence of a tissue to respond to organizer signals, and thus emphasize the need to focus on the nature of this responsiveness. Together, our arguments call into question the universality of the organizer concept as a mechanism to establish the principal body axes during early vertebrate embryogenesis.

## Fate maps and body plans

**Fig. 1.**
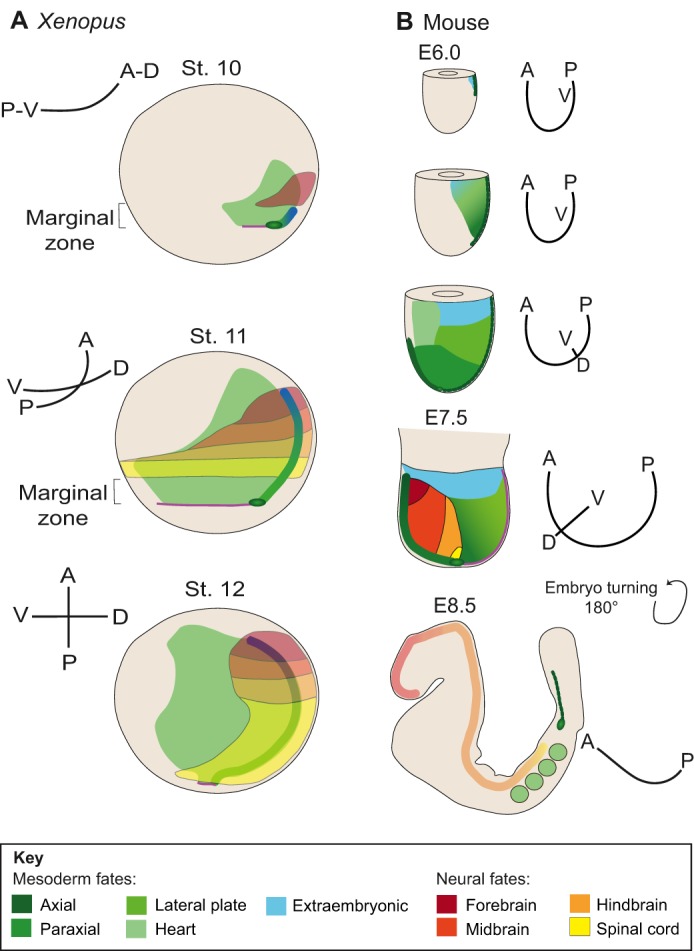
**Unfolding of the principal body axes during gastrulation in *Xenopus* and mice.** (A) *Xenopus* embryos at progressive developmental stages (st. 10-12) positioned with the blastopore lip on the right and the animal pole to the top. The prospective neural plate is shown in colours ranging from red (anterior) to yellow (posterior). Involuted mesodermal tissue is shown in green. Non-involuted mesodermal tissue is not shown. (B) Progressive emergence of the body axes in mouse. Mouse embryos are depicted at progressive stages of gastrulation, with proximal to the top and distal to the bottom. Lateral view, with the approximate future axes shown on the right. Note two important features of mammalian gastrulation, namely the impossibility of mapping the dorsoventral (DV) axis onto early gastrula stage embryos, and the progressive increase in the size of the embryo during gastrulation (not to scale). In both species, prospective head mesodermal cells arise from the non-involuted marginal zone/primitive streak and move anteriorly as anterior neural tissue is becoming specified in the overlying ectoderm/epiblast.

Much of our understanding of developmental biology is built on the experimental embryology of the amphibian embryo and, for this reason, its fate map has become a guide for understanding the unfolding of its body plan and that of other organisms (Wolpert et al., 2015), and for interpreting the role of the molecular networks associated with this process. A key feature of this fate map is the definition of the meridian as the dorsoventral (DV) axis, with Spemann's organizer arising at the prospective dorsal side (Dale and Slack, 1987). Such a map is difficult to reconcile with that of gastrula stage amniote embryos, whose main reference is an anteroposterior (AP) axis. However, between 2000 and 2006, a series of experiments, combined with a critical analysis of the classical literature, led M. C. Lane and M. D. Sheets to propose a revision of the classical amphibian fate map (Lane and Sheets, 2000, 2002a,b, 2006). An important element of this work was the labelling of individual blastomeres at the 32-cell stage and allowing development until later stages than in other experiments to follow their contributions to the body plan. This led the authors to relabel the DV axis as the AP axis, with the prospective DV axis running along the animal-vegetal axis. Surprisingly, with very few exceptions (Gerhart, 2002; Kourakis and Smith, 2005; Kumano and Smith, 2002; Meinhardt, 2006), these studies have been either ignored or glossed over as a simple footnote to what was already known (Harland, 2008). However, it is clear that, at the very least, they highlight the need for a careful assessment of fate maps to interpret experiments (Gerhart, 2002; Lane and Sheets, 2006). Perhaps this attitude is derived from the consideration that analyses of fate maps at the 32-cell stage offer a limited resolution of how the future body axes are mapped onto the gastrula stage embryo, since the axis can be divided into 32 differently labelled territories at the most. Furthermore, it is known that additional movements, such as vegetal rotation, occur between this stage and the onset of gastrulation movements at the blastopore lip, and thus can distort the relative position of cells (Winklbauer and Damm, 2012; Winklbauer and Schurfeld, 1999). However, fate-mapping experiments performed at a later time, stage 10, with vital dye labelling (Keller, 1975, 1976; Steventon et al., 2009; Steventon and Mayor, 2012) also support and refine the observations of Lane and Sheets. Together, these studies reveal that, at the onset of gastrulation, future anterior structures map together with future dorsal structures, and that these axes run down towards a future ventroposterior region opposite to the blastopore lip (summarized in Fig. 1).

**Fig. 2.**
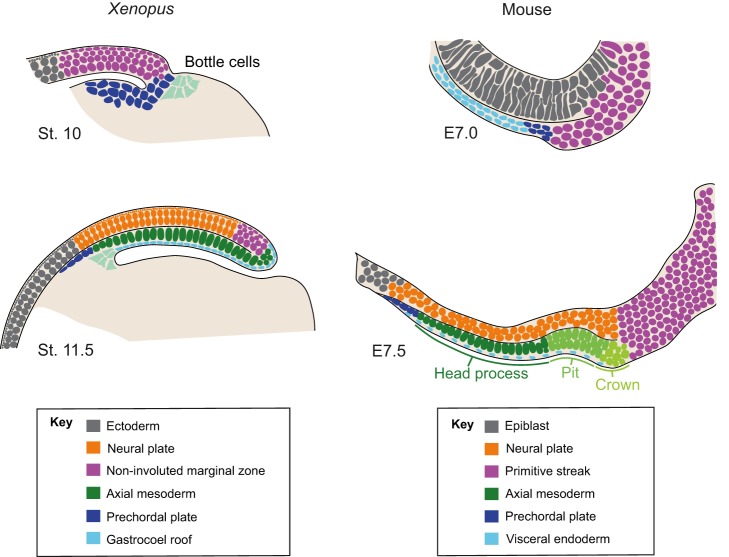
**Similarities between the mouse node and the *Xenopus* blastoporal lip.** Diagrams depicting sagittal transverse sections through the *Xenopus* blastoporal lip region (left) and the mouse node (right) at successive stages of gastrulation. Cells are colour coded to highlight homologies between tissues and their fates (see key). Species-specific structures are colour coded and labelled on the figure. These include bottle cells in *Xenopus* that drive the primary invagination of cells at the blastopore lip, and an epithelial indentation in the mouse node region, often referred to as the ‘pit’, that has motile cilia and acts as the source of the prechordal plate and the notochord. Posterior to the pit is a bulging structure often referred to as the ‘crown’, which gives rise to the postanal component of the notochord. Note that because of these two related structures, there is often some confusion in the literature as to what is considered the ‘node’ proper; the term has been used to refer to either the pit or both the pit and the crown. Here we use the term ‘node’ to refer to both structures, which are often transplanted together.

The fate map resulting from these observations remains an oversimplification but emphasizes the difficulty of implementing a simple Cartesian mapping of body axes onto the gastrula stage embryo (for example, see Niehrs, 2010). Importantly, it allows for an easier comparison with amniote fate maps which, either in a disc or cylinder arrangement, have the AP axis as their main reference (see also Stern et al., 1992). With this in mind, from the perspective of trying to better understand Spemann's organizer, we propose that the most appropriate comparison in terms of fate maps across species is a correspondence between a frog at the onset of gastrulation (stage 10) and a mouse embryo at the end of primary gastrulation [embryonic day (E) 7.5]. The main reason for our suggestion derives from the observation that the first cellular activity stemming from the organizer, namely the extension of the head process and the prechordal plate, can be observed in the mouse embryo after the appearance of the node at E7.5 (Fig. 2).

The different interpretations of early fate mapping data in *Xenopus* highlight an important issue regarding the reading of this information during gastrulation, namely that there is no simple way to map later embryonic axes onto a common plan of the pre-gastrula embryo. The inherently dynamic nature of the way the body axes unfold during gastrulation, and the associated variety of gastrulation modes, requires a precise analysis of when and where signalling and responding centres are in contact. This is especially important when seeking to identify structures homologous to the Spemann organizer in other species such as the mouse. For example, a very basic comparison might suggest that the Spemann organizer at the onset of gastrulation is equivalent to the node of the mouse at the completion of primary gastrulation (Fig. 2). However, as we discuss below, there are problems with drawing direct structural and functional homologies between these two structures.

## On the universality and diversity of the organizer concept

In mammals, the node is a well-defined structure that has an established and accepted function as the origin of the prechordal plate and notochord, and also plays a crucial role in specifying left-right (LR) asymmetry (Blum et al., 2007; Hirokawa et al., 2006; Lee and Anderson, 2008; Shiratori and Hamada, 2006). In transplants between mouse embryos, this structure is capable of inducing postoccipital axial structures, which has been interpreted to be a manifestation of partial organizer activity (Beddington, 1994; Tam and Beddington, 1992). Whether this is sufficient evidence to consider the node a homologue of Spemann's organizer is a matter for discussion. The argument is based on the observation that in xenotransplants into chickens and frogs, the mammalian node has the ability to induce anterior structures (Blum et al., 1992; Kintner and Dodd, 1991; Knoetgen et al., 2000). However, we believe that these observations reflect the state of the responding tissue rather than the potency of the transplanted tissue (see below) and, for this reason, we rather abide by the definition implicit in the original Spemann and Mangold experiment that the activity of the organizer should be defined in an allotransplant test.

In anamniotes, there is a clear separation between LR patterning and the Spemann organizer. In *Xenopus*, the structural and functional homologue of the node is the gastrocoel roof plate (GRP), a ciliated structure that emerges at the posterior end of the embryo by mid-neurulation, in connection with LR patterning, at a time when the organizer has lost significant inducing ability (Blum et al., 2007; Shook et al., 2004; Walentek and Quigley, 2017). A similar structure, termed Kupffer's vesicle, can be found in zebrafish at an equivalent time in development and is separate from the organizer (Essner et al., 2005, 2002). Thus, as the amphibian equivalent of the node does not have organizer activity, and as the mammalian node does not have the functional properties of the amphibian organizer, it is misleading to conflate the two [for some of the historical reasons for the reinforcement of the two, see Blum et al. (2007)]. A crucial difference between the two structures is made most obvious when one considers that an important function of the organizer is to promote the specification of anterior neural identity in allotransplants, whereas the node of mouse embryos has very limited anterior neural induction ability, and the loss of the node has no effect on the emergence of neural tissue (Ang and Rossant, 1994; Davidson et al., 1999; Dias and Schoenwolf, 1990; Liguori et al., 2003; Storey et al., 1992; Weinstein et al., 1994).

Birds appear to have features of both amphibians and mammals and, in this sense, Waddington's speculation might be partially correct. For example, in the manner of mammalian embryos, chicks have a node that harbours axis-inducing ability in allotransplants (Dias and Schoenwolf, 1990; Storey et al., 1992). The chick node emerges at the most anterior position of the primitive streak, which will become the boundary between the hindbrain and the spinal cord, i.e. a region homologous to that where the node appears in the mammalian embryo. However, in contrast to mammals, the leading edge of the advancing chick primitive streak contributes to the node and has neural- and axial-inducing features (Bachvarova et al., 1998; Dias and Schoenwolf, 1990; Izpisua-Belmonte et al., 1993; Storey et al., 1992) and thus can be considered an intermediate between the full inducing potential of Spemann's organizer and the distributed situation seen in mouse embryos.

Altogether, and with a stringent definition of Spemann's organizer, these observations suggest that it is difficult to establish homologies between the node and the amphibian organizer. As we explore below, this raises the question of how we can better understand the relationship between these structures.

### Spemann organizer functions are spatial and temporally distributed in other model organisms

Vertebrate embryos demonstrate a remarkable diversity in the timing of developmental events during early embryogenesis, as well as in their growth rates and geometrical constraints (Steventon et al., 2016; Steventon and Martinez Arias, 2017). Therefore, the degree to which a single signalling region is able to organize the formation of an entire secondary axis may depend on species-specific differences in the timing of cell specification events, and the relative positioning of signalling and receiving tissues. Indeed, experiments in amphibians in which organizers of different ages are transplanted into host tissues revealed that their inducing capacity changes its potential with age. In particular, an organizer can be shown to lose the ability to induce anterior structures over time and, instead, to increase its ability to induce more posterior structures and to self-differentiate (Mangold, 1933; Nieuwkoop, 1997). These observations led to the notion of the existence of distinct ‘head’ and ‘trunk’ organizers that can be separated spatially and temporally (Stern, 2001; Stern et al., 2006). Importantly, whereas in most amphibian experiments the head organizer exhibits continuity with the tail organizer, i.e. transplants of an organizer at an early stage can elicit a complete axis, in the case of mammals there is no single entity that will yield the complete axis (Kinder et al., 2001). Therefore, the close spatial and temporal coincidence of head and trunk organizers could be a characteristic of amphibian embryos, with distinct functionalities of the organizer being separated in space and time depending on the species in question.

A search for mammalian organizers analogous to that of amphibians led to the identification of transient groups of cells during gastrulation, each of which provides partial organizer functions. In these experiments, the transplantation of temporally separated pieces of primitive streak into uncommitted regions of the epiblast revealed an ability of cells from different tracks of the primitive streak to perform partial functions as ‘head inducers’. This led to the notion that, in the mouse, an equivalent of Spemann organizer function is spread across three populations: the early gastrula organizer (EGO), the mid-gastrula organizer (MGO) and the node, which acts as an independent ‘trunk’ organizer (Kinder et al., 2001; Tam and Steiner, 1999). Furthermore, in an experimental tour de force, Tam and colleagues showed that only the juxtaposition of cells from the anterior visceral endoderm (AVE), the epiblast and the MGO creates a structure that behaves, at a low frequency, as the Spemann organizer (Kinder et al., 2001).

The notion of a ‘head organizer’ in mice is problematic, but it is agreed that, if it exists, it is associated with the AVE (Beddington and Robertson, 1998, 1999), an extraembryonic structure that emerges before gastrulation (Takaoka and Hamada, 2012). However, although this structure can ‘induce’ anterior neural markers, and in some instances a whole axis, in chicken embryos (Knoetgen et al., 2000), it cannot do this when transplanted between embryos in mice, where it is not sufficient for the development of anterior neural structures. In avian embryos, the hypoblast can induce anterior neural character (Eyal-Giladi and Wolk, 1970; Foley et al., 2000) and can be considered homologous to the mouse AVE (Stern and Downs, 2012).

Differences in the morphogenetic processes that drive gastrulation are likely determinants of these shifts in the spatiotemporal separation of organizer functions, and several recent studies have discussed how these transformations might have occurred in vertebrate evolution. Amniote gastrulation through a primitive streak has been well described via live imaging studies of the chick embryo, and the transition from an amphibian-like convergence extension process has been discussed in relation to planar cell polarity signalling (Voiculescu et al., 2007). Furthermore, a comparison with gastrulation in reptiles highlights the flexibility in the morphogenetic processes that drive gastrulation across amniotes and even vertebrates (Bertocchini et al., 2013; Stower and Bertocchini, 2017). Indeed, chondrichthyan dogfish display aspects of gastrulation that are similar to that of both amniote and anamniote species, despite their evolutionary position basal to both teleosts and amphibians (Sauka-Spengler et al., 2003). Such alterations in morphogenesis are likely to impact the way in which organizer functions are modularized during evolution. These differences are in turn associated with alterations in the modes of nutritional supply to the embryo, whether it be through viviparity in the case of mammals, meroblastic cleavage above a large yolk in the case of avians, reptiles and chondrichthyans, or through holoblastic cleavage in the case of amphibians.

Species-specific differences in the geometry of embryos around the time of gastrulation may also influence how they organize their axes. For example, fish embryos undergo meroblastic cleavage and gastrulate by completely enclosing their yolk sac, and offer a good example of how organizer function can be distributed in space as compared with amphibians. In zebrafish, the embryonic shield is situated in the dorsalmost region of the marginal zone and can induce ectopic head and trunk structures upon transplantation (Shih and Fraser, 1996). Conversely, transplantation of the ventralmost region of the marginal zone elicits only tail structures (Agathon et al., 2003), whereas intermediate regions of the marginal zone can induce trunk and posterior head structures (Fauny et al., 2009). More recently, it has been demonstrated that the entire spectrum of zebrafish organizer functions can be recapitulated by opposing sources of Nodal and BMP signals (Xu et al., 2014). During normal development these events occur almost simultaneously during the spreading of the embryonic tissues over the yolk sac. Thus, unlike the temporal separation that is observed for head, trunk and tail specification events in mouse embryos, zebrafish embryos have a spatially distributed organizer that is a likely consequence of differences in the morphology of the early gastrula. Indeed, slight alterations in yolk size can drastically alter the timing of developmental events, as has been observed in trout embryos (Finch et al., 2010).

**Fig. 3.**
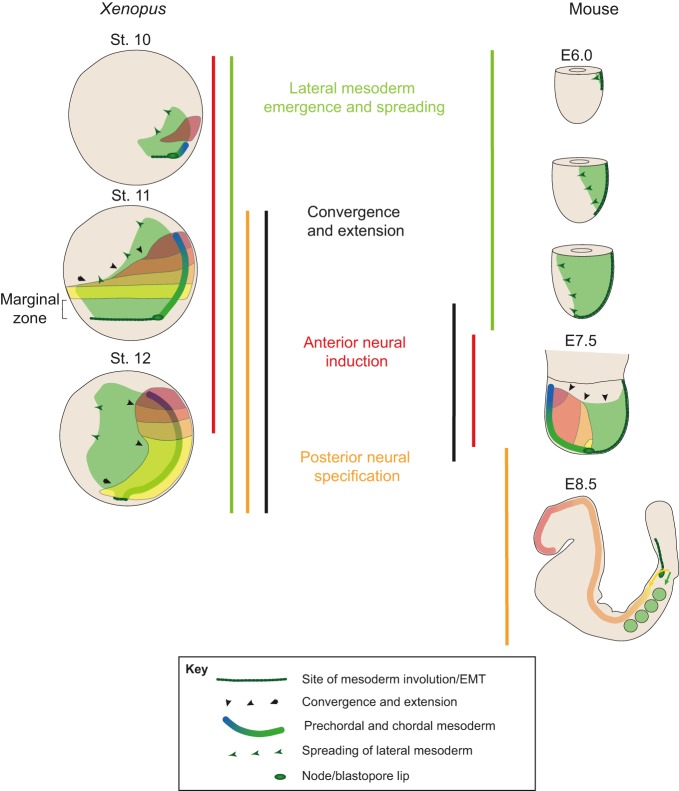
**Heterochrony in gastrulation processes between mouse and *Xenopus* embryos.** The diagrams on the left show *Xenopus* embryos at progressive stages of gastrulation with the prospective anterior to the left and posterior to the right. On the right are diagrams of mouse, shown with anterior to the left and posterior to the right, to follow convention. In both cases, the prospective neural plate is shown in colours ranging from red (anterior) to yellow (posterior). Involuted mesodermal tissue is in green. Non-involuted mesodermal tissue is not shown. Coloured bars indicate the principal cellular processes associated with gastrulation and how they map to different stages when comparing the two species. Note how these processes overlap in time in *Xenopus* but are temporally separate in mouse. EMT, epithelial-mesenchymal transition.

Overall, these observations suggest that the activity of the organizer is dispersed in time and space to differing degrees depending on the organism in question. This is taken to an extreme in the case of the mouse embryo, where the gastrulation processes regulated by the organizer are themselves spread out spatially and temporally as compared with *Xenopus* (Fig. 3). Furthermore, the organizer in amphibian embryos has a spatial molecular organization, with groups of cells expressing specific transcription factors and signals that become allocated to defined cell types as this ensemble unfolds spatially during the process of gastrulation (Harland and Gerhart, 1997; Vodicka and Gerhart, 1995; Zoltewicz and Gerhart, 1997). This contrasts with mouse embryos in which this cellular and molecular ensemble emerges following a temporally organized gene expression programme (Kinder et al., 2001; Tam and Gad, 2004; Tam et al., 1997). These observations suggest that it might not make sense to search for a region of the mammalian embryo that can elicit all the aspects of the amphibian organizer, as the responses to organizer function are themselves spread out in space and time, in a species specific manner. Thus, rather than dubbing cell populations with different ‘organizer’ functions, we should look at them as sources of different molecules that vary from species to species.

### Species-specific differences in competence determine organizer action

The focus on the activity of organizers has also obscured the fact that there are species-specific differences in the way tissues respond to node transplants. Most surprising is the finding mentioned above that, whereas a mouse node is incapable of inducing a complete axis when transplanted into another mouse embryo (Beddington, 1994; Tam and Beddington, 1992), it can elicit a ‘full organizer response’ when transplanted onto the zona opaca of a chicken embryo at the appropriate stage (Kintner and Dodd, 1991; Knoetgen et al., 2000). Furthermore, the node of the mouse will elicit anterior neural tissue as well as convergence and extension movements upon contact with animal cap tissue from *Xenopus* (Blum et al., 1992). To understand this further, we need to appreciate that the outcome of an organizer experiment depends very much on matching the age of the graft with that of the host, i.e. the experiment requires the precise tailoring of the ages of the participant tissues and a species-specific adjustment in order to obtain the right answer. Given that the same ‘organizer’ can elicit different responses in different species, this leads to the conclusion that the response to the transplant is highly dependent on the state of the host tissue.

The ability of a tissue to respond to the organizer is often referred to as its ‘competence’ (Christian and Moon, 1993; Grainger and Gurdon, 1989; Servetnick and Grainger, 1991). This notion became paramount in the interpretation of organizer experiments early on (Hamburger, 1969), leading J. Holtfreter to state that ‘the organizer tissues do not actually organize the cell material whose new trend they have induced. Rather the induced cells organize themselves into complex organs…in later discussions on this issue I went as far as to declare the term organizer to be a misnomer’ (Holtfreter, 1985). Thus, the emphasis on the organizer, whatever its origin and composition, as the source of the responses will always miss the important point that its action is non-specific and is completely dependent on the state of the host at the moment of the transplant. This was noticed most clearly by C. H. Waddington, who discussed the problem in terms of the organizer ‘evocating’, rather than ‘inducing’, a response in the host (Waddington, 1954), i.e. the organizer does not create a new state but rather brings out a response that is latent in the host tissue at the moment of the transplant. From this perspective, it is clear that frog, fish and chicken embryos have, at a certain moment early in development, a very broad competence that allows them to respond to the signals from organizers of different species.

### Cell numbers and competence in mammalian embryos

One possible explanation for the restricted competence of the mouse embryo might lie in the process of unfolding of the fate map of mammalian pre-gastrulation embryos. When comparing the emergence of the body plan during gastrulation in amphibians and mice, there is a difference that we believe is important and has been overlooked, namely the cellular mass that is available for the process. At the onset of gastrulation, the mammalian embryo is an epithelium that, depending on the species, contains only 400-600 cells (Snow, 1977) in comparison with the thousands of cells found in an equivalently staged amphibian embryo, i.e. the mammalian embryo at this stage does not have enough cells to accommodate the numbers that will lay down the body plan. Although it is possible to outline a fate map of the epiblast, it is a highly plastic map with very little geographical determination. Thus, whereas amphibian and fish gastrulation is, primarily, a process of cell redistribution with little proliferation in a constrained volume, gastrulation in mammals is associated with a large increase in cell numbers and volumetric growth (Lawson and Pedersen, 1992; Smith et al., 1994). This consideration of the numbers of cells that are available for gastrulation also applies to the chicken embryo, which is composed of thousands of cells before gastrulation and in which gastrulation redistributes these cells. In this sense, chicken and amphibian embryos are very similar to each other. In both cases, the cells that give rise to the fore-, mid- and hindbrain exist before gastrulation and are placed into position by the movements associated with gastrulation. Thus, in the chick, the leading edge of the primitive streak can be fate mapped to the start of gastrulation (Koller's sickle), as is the case for the leading edge of involuting bottle cells in *Xenopus* (dorsal lip) (Lawson and Schoenwolf, 2001, 2003). However, this is not possible in the mouse embryo (Kinder et al., 2001).

Given that mouse embryos begin gastrulation with considerably fewer cells, it is not surprising that formation of the primitive streak in mice results from the convergence of three processes: a growth process during which progenitor cells of the future body axis are being progressively added; a specification process in which cells are assigned distinct fates; and a morphogenetic process (Lawson and Pedersen, 1992; Smith et al., 1994). Thus, while different regions of prospective mesoderm can be mapped onto the chicken embryo during primitive streak formation (Camp et al., 2012; Garcia-Martinez et al., 1993; Hatada and Stern, 1994; Iimura et al., 2007; Psychoyos and Stern, 1996; Selleck and Stern, 1991), this is much more difficult to achieve in the mouse, where cells are being continually added (Lawson and Pedersen, 1992; Smith et al., 1994). These observations not only emphasize the differences between embryos of different species but also highlight the difficulty associated with generating fate maps at early stages of mouse development, as the primordia of the structure that is being fate mapped might not exist at the time of the experiment.

## Conclusions and perspectives

Our considerations on the structure of Spemann-like ‘organizers’ in amphibian and mammalian embryos, their relationship to the node and to the pre-gastrulation fate maps, and the appreciation that different species have different cell numbers available for morphogenesis at the time of gastrulation, lead us to five conclusions.

First, pre-gastrulation fate maps are difficult to establish with detail and precision and to translate across species. Notwithstanding this, we recognize that a slight modification of the Lane and Sheets revision of the amphibian embryo fate map generates a useful frame of reference that allows the comparison of pre-gastrulation embryos of different species and reveals the existence of heterochronies and heterotopographies but also conserved modules. Second, a simple structural and functional relationship between the node and the organizer is untenable. Third, the amphibian organizer, which originates the concept, is a contingent collection of elements, each with a specific function that unfolds over time during gastrulation; it is a structure characteristic of amphibian embryos. In other organisms, however, the same elements are dispersed in time and space to different degrees and this makes it difficult to talk about a universal ‘organizer’ in the sense originally described by Hans Spemann. Fourth, the term ‘organizer’, if understood as a single signalling region, is not helpful. The notion of an organizer refers to specific experiments that test the signalling ability of specific groups of cells in particular contexts. The use of the term ‘organizer’ should therefore be restricted to the outcome of precise experiments: a heterologous allotransplant in the same embryo, similar to that performed by Spemann and Mangold. Any other use of the term, particularly in transplants between species, is testing the ability of the graft to signal, as well as that of the host to respond. Finally, in our view, knowledge of the state of the host or responding tissue is essential for the interpretation of an organizer grafting experiment.

Although some of these considerations came to the fore in the years following the discovery of the organizer (Hamburger, 1969; Holtfreter, 1985) and might be considered ‘common wisdom’, their impact in the analysis of organizer effects is often minimized in favour of the apparent instructive potential of the grafts. This feature is most clearly demonstrated in experiments in which, for example, a node is transplanted to the anterior region of a limb bud and is shown to induce digit duplications (Hornbruch and Wolpert, 1986); this is not because the node is a ‘digit organizer’ but because it expresses Shh which, in the context of the early limb bud, will specify digits. Given that a bead soaked in Shh can lead to the same outcome, this finding highlights that an inducer can also produce non-specific signals that elicit a tissue-specific response. In the terminology of Waddington, organizers ‘evoke’ responses that are latent in the responsive tissue. We therefore surmise that the key to understanding the activity of an organizer does not lie in what it does, but in the competence state of the responsive tissue. Overall, and as we discuss below, these five key conclusions have important implications for future studies and analyses, and also highlight several open questions in the field.

### Implications of a competence-focussed understanding of organizer action

Although it is clear that, in the original Spemann and Mangold experiment and in the variations that followed, the organizer induces an axis that includes mesodermal and neural derivatives, Spemann's organizer is most commonly associated with the process of neural induction: the generation of a neural plate from the ectoderm, where it is thought to ‘instruct’ this fate on an ectodermal primordium (Andoniadou and Martinez-Barbera, 2013; De Robertis and Kuroda, 2004; Stern, 2005). The molecular underpinning of this event is tightly linked to the activity of BMP, which was first shown to suppress neural fate in *Xenopus*. For example, the organizer acts as a timed and spatially localized source of antagonists for BMP, Nodal and Wnt signalling, which later were also shown to be involved in the control of neural fate (reviewed by De Robertis, 2009; De Robertis et al., 2001; Harland and Gerhart, 1997; Stern, 2005). The observation that the AVE and the node of amniote embryos also express these inhibitors suggested a universality of the process. However, as with many of the issues raised above, there are multiple exceptions to the rules implied in the *Xenopus* experiments (Stern, 2005). For example, whereas BMP is necessary and sufficient to inhibit neural fates in mammalian embryos (Di-Gregorio et al., 2007; Malaguti et al., 2013; Zhang et al., 2010), it is not sufficient in chicken embryos (Linker and Stern, 2004; Streit et al., 1998). A possible explanation for these differences might derive not only from a consideration of the role that additional signals might play in the process (Stern, 2005; Wilson and Edlund, 2001) but also from a refocus of the interpretation of these experiments on the competence of the host tissue.

Our view underpins the notion that the epiblast of vertebrate embryos has a default fate as anterior neural tissue (Hemmati-Brivanlou and Melton, 1997, 1994; Levine and Brivanlou, 2007; Muñoz-Sanjuán and Brivanlou, 2002; Stern, 2005). However, rather than a default fate, which can imply the passive acquisition of a fate, we suggest that the epiblast is ‘primed’ for an anterior neural fate through the activity of a specific intracellular gene regulatory network (GRN) that is active in all cells of the epiblast at the onset of gastrulation. The activity of this network is likely to be suppressed or modulated by BMP, and the events associated with neural induction relate to the spatial and temporal modulation of this programme. In this sense, we believe that organizer signals are not an integral component of this primary GRN, but rather are part of a parallel network and act to evoke the activity of the autonomous GRN. At the tissue level, signals act as selectors for either default or alternative fates and will ultimately determine the proportions of cells that enter one state or another. We suggest that this is universal and that embryos from different species exhibit different degrees of sensitivity to BMP and its antagonists in the process of triggering or allowing the activity of these autonomous GRNs: in the context of neural induction, competence could thus be understood as the complexity or responsiveness of this primary proneural GRN. It will be important to understand the molecular basis of these different sensitivities but we believe that this suggestion provides an explanation for why presumptive mouse organizers can do more in a chicken embryo than in a mouse embryo: the competence of the two tissues to the same signal is different, i.e. both the degree of activity of the preneural GRN and the thresholds required to evoke the GRN differ between chicken and mouse.

The notion of a primary epiblast fate with an anterior neural character is further supported by the analysis of mouse mutants for BMP and Nodal (Camus et al., 2006; Di-Gregorio et al., 2007), which develop an anterior neural fate in the absence of mesoderm, as would be predicted by the default hypothesis. In *Xenopus* embryos, anterior neural fate can be elicited in the absence of mesoderm but, as in the mouse, even this requires BMP inhibition (Kuroda et al., 2004). This situation can also be observed in chicken, where the requirement for the suppression of BMP signalling is associated with transcriptional regulation but before the onset of gastrulation (Wilson et al., 2000), which thus provides an explanation for the lack of an effect of BMP inhibition after the onset of gastrulation, i.e. the competence of the tissue has changed. Furthermore, direct evidence for the existence of an underlying GRN associated with anterior neural fate can be found in the transcriptional pre-pattern of this region in mouse and amphibian embryos, i.e. the localized expression of transcription factors of, for example, the Iroquois family (reviewed by Andoniadou and Martinez-Barbera, 2013; Bainter et al., 2001), the activity of which is modulated by BMP.

This consideration of BMP signalling as a modulator of the dynamics of the GRNs that are active in the epiblast is not far from the views expressed by Lane and Sheets (Lane and Sheets, 2006; see also Malaguti et al., 2013), who suggested that ‘the frog blastula/gastrula uses BMPs as a widespread repressive mechanism to maintain and protect a reservoir of quiescent cells, and the BMP antagonists released by the organizer ensure an orderly, progressive entry of a few dorsal mesodermal cells at a time so that the body plan is completed when the reservoir is emptied’. Rather than a quiescent state, we suggest that the host tissue is in a ‘genetically primed’ state.

Altogether, these observations suggest a reformulation of neural induction, and thereby of the activity of Spemann's organizer, as the degree to which an intrinsic, and perhaps universal, genetic programme is ‘evoked’ and the molecular mechanisms associated with this process. The question now relates to the mechanisms associated with this evocation process, i.e. how does the activity of BMP control the dynamics of these anterior neural GRNs? Furthermore, if induction is actually an evocation (Waddington, 1954), this would imply that transplantation of the dorsal lip to the prospective posterior/ventral side in the Spemann and Mangold experiment should not be interpreted as the establishment of a new programme of gene expression in the host but rather as the unveiling of a latent programme. A similar interpretation was put forward by Lane and Sheets with some experimental support (Lane and Sheets, 2006). One of the arguments that they provided is the observation that the marginal zone of the amphibian embryo is already specified (i.e. it expresses T/Bra and MyoD) and during gastrulation converges and extends to form the postoccipital region. Gastrulation activates a programme of differentiation that is suppressed by BMP in the posterior side. The organizer releases, in an ordered manner, the BMP and Wnt antagonists and elicits these programmes of differentiation to give rise to mesodermal and ectodermal derivatives that are progressively patterned along the AP axis. In support of this, it has been shown that BMP signals act to reserve a population of mesoderm progenitor cells for entry into the tail mesoderm in zebrafish (Szeto and Kimelman, 2006). What this explanation lacks is an understanding of the molecular networks that maintain cells in an uncommitted progenitor state, and of how this is released upon BMP inhibition. Despite the importance of the molecular mechanisms that act to control the timing of competence and determination, and the recent progress in insight into the molecular epigenetic mechanisms that could act to control this, little has been done to link these mechanisms explicitly to the early patterning events that drive organizer function and the response of tissues to its signals.

### Open questions and future perspectives

Our analysis of the timing of organizer function in relation to the appearance of the node and to the fate map at the onset of gastrulation in the principal model organisms suggests that the relative timing of developmental events in different tissues is of great importance when considering the composition and roles of an organizer or organizer-like tissue. The modularity of the organizer has allowed for, and might explain, the great evolutionary flexibility in the relative timing of specification events, as well as differences in the spatial positioning of signalling and responding tissues. This has been essential in allowing for diversity in maternal-embryo trade-offs during the evolution of vertebrates. We suggest the need for a shift in the understanding of organizer function away from the graded release of instructive signals and towards understanding the timely release of signals that act to evoke autonomous GRNs. This probably accounts for how evolutionary changes in timing, morphology and growth can be accommodated because they feed back onto the timely release of conserved developmental trajectories via signalling. In this sense, we pose that pattern formation should not be seen as a downstream output of organizers and their responding tissues, but rather as an emergent property of their dynamic interaction. These changes must then be canalised during evolution, in order to obtain robust and reproducible development. We argue that this largely depends on tuning the windows of competence within which responding tissues can or cannot respond to organizer signals.

Moving forward, there are several questions that need to be addressed. For example, what is the fine structure of the GRNs that underlie the default pre-proneural fate? Are there, as surmised above, signals that control dynamic aspects of these GRNs and, if so, how do they achieve this? Obtaining the answers to these questions will require an understanding of the relationship between GRNs and competence over time, and how this varies across species. We will also need to relate these GRNs and the processes that they govern to an understanding of precisely when and where cells undergo cell state transitions during normal development, and how this corresponds to their spatiotemporal exposure to specific signals. Overall, such a systems-level understanding of these processes will shed light on the degree of regulative ability inherent within a given embryo and, ultimately, how this confers adaptability to changes in embryo size and developmental rates during evolution. Much of this work will demand detailed analyses of the transcriptional and epigenetic mechanisms operating in developing tissues with high spatial and temporal specificity.

While the ultimate challenge will be to map these events back to the whole embryo, it is clear that much can be gained from studying *in vitro* systems that use embryonic stem cells (ESCs). Over the last few years, ESCs have emerged as a versatile experimental system for studying developmental events that are less accessible to experimentation and live imaging over extended periods of time, such as those occurring in mammals (Keller, 2005; Turner et al., 2016). From the perspective of this discussion, a number of experiments have shown that ESCs have an intrinsic primary anterior neural fate that can be externally modulated and experimentally probed (Gouti et al., 2014; Malaguti et al., 2013; Turner et al., 2016, 2014), thus providing additional support for the notion of a primary proneural fate of the vertebrate embryo. Furthermore, a significant value of ESCs is, and will be, in the study of human development. In this regard, there are reports of the derivation of human organizers from human ESCs (Martyn et al., 2017 preprint; Sharon et al., 2011). These findings provide the first opportunity to test within a human framework what we have learned in the embryos of model organisms. However, as we have suggested here for the xenotransplantation of mouse node tissues, such experiments might be revealing more about the competence of the host tissue rather than the inducing properties of the donor tissue itself. Nonetheless, these studies open up possibilities to perform similar embryological experiments in humans to those that have been so informative within established developmental model organisms. In this sense, we might be witnessing a revitalization in the field of experimental embryology.
